# Operation of a giant occipital encephalocele in an infant: A surgical case report

**DOI:** 10.1016/j.ijscr.2024.109681

**Published:** 2024-04-27

**Authors:** Hamide Barzegar, Marzieh Davoodi, Shahnaz Pourarian, Hamid Reihani

**Affiliations:** a Pediatric Department, Neonatal Research Centre, Shiraz University of Medical Sciences, Shiraz, Iran; b Student Research Committee, Shiraz University of Medical Sciences, Shiraz, Iran; c Neonatal Research Centre, Shiraz University of Medical Sciences, Shiraz, Iran; d Student Research Committee, School of Medicine, Shiraz University of Medical Sciences, Shiraz, Iran

**Keywords:** Encephalocele, Infant, Surgery case report

## Abstract

**Introduction and importance:**

Encephalocele is a rare medical condition where certain parts of the central nervous system protrude through a skull defect, resulting in a deformity where the head size is smaller than the protrusion. This condition is relatively uncommon, and only a few cases have been reported worldwide.

**Case presentation:**

We present a case of a 13-day-old neonate with a giant occipital encephalocele who underwent a successful surgical intervention in a resource-limited setting.

**Clinical discussion:**

The diagnosis of encephaloceles is frequently by clinical examination, although sonography could be helpful before birth. It is crucial that this patient receives immediate surgical intervention. In cases where hydrocephalus and ventriculomegaly are absent, we predict a better prognosis. The prone position is preferred in these operations, and Anesthesia is a real challenge.

**Conclusions:**

Congenital giant occipital encephaloceles can be identified clinically shortly after birth. They cause a substantial surgical challenge due to their massive size. Surgical repair must be performed as early as possible.

## Highlights

• An encephalocele, a type of neural tube defect, is the herniation of intracranial contents through a bone defect.
• The most effective method for treating encephalocele is surgery, and the ideal window of time for this procedure is from birth to four months of age
• Treating a giant encephalocele is challenging, and we chose the prone position for the operation as it is the most suitable for such cases due to better maneuverability.

## 1 Introduction

An encephalocele, a rare type of neural tube defect, is the herniation of intracranial contents through a bone defect [1,2]. The term giant encephalocele is used when the head size is smaller than the encephalocele [3]. Encephaloceles are rare, with an incidence of about 1 in 5000 live births [5], varying based on geographical location and race [6]. Depending on the area involved, they can be divided into anterior and posterior variants [7]. Encephalocele in children is usually congenital, and in adults, it can be traumatic or iatrogenic. [[4], [5], [6]] The location, size, and nature of the brain tissue protruding through the bony defect, as well as any cerebrospinal fluid circulation problems, can significantly affect the symptoms of this disorder [7,8]. Occipital encephalocele is the most common of all encephaloceles [8]. The most effective method for treating encephalocele is surgery; the ideal window period for this procedure is from birth to four months of age [8]. In this article, we report a 13-day-old case with a giant occipital encephalocele. Our article has been reported in line with The Surgical Case Report (SCARE) 2023 guideline [9].

## 2 Case presentation

The patient was a male neonate delivered by Caesarean section at a gestational age of 39 weeks from a 27-year-old woman. His first-minute Apgar score was nine. His parents took him home despite the presence of a huge sac on the occipital region. After 13 days, he developed poor feeding and lethargy, and the parents brought him to our center.

Upon admission of the 13-day-old boy, the vital signs and growth parameters were as follows: pulse rate: 130/min, respiratory rate: 50/min, blood pressure: 80/50 mmHg, temperature: 37 °C. His weight was 3000 g. His head circumference was 33 cm, height was 48 cm, and anterior fontanelle was 2 × 2 cm. On physical examination, he had poor sucking. The Moro reflex and deep tendon reflexes (DTRs) were normal. On the posterior occipital region, we observed a huge swelling covered by intact skin, larger than his head, it measured approximately 18.8 × 10.5 × 6.1 cm. His skin was warm and dry, and there was no sign of petechiae or purpura. His lung was clear in auscultation and had symmetrical expansion. And normal heart sounds were detected. According to laboratory data, he had high BUN, sodium, and potassium due to dehydration which improved during the course of hospitalization (Table 1).

**Table 1:** Laboratory data of the patient.

Lab data	Day 0 (Admission)	Day 1	Day 2	Day 4
White blood cells (/mm^3^)	11,600	–	–	12,000
Hemoglobin (gm/dL)	20.8	–	–	15.9
Mean corpuscular volume (fl)	105.4	–	–	99.6
Platelets (/mm^3)^	527,000	–	–	203,000
Prothrombin time (s)	16.2	–	–	–
International normalized ratio	1.2	–	–	–
Partial thromboplastin time (s)	31.8	–	–	–
Blood urea nitrogen (mg/dl)	41	17	11	4
Creatinine (mg/dl)	0.41	0.27	–	0.19
Sodium (mEq/l)	155	146	139	142
Potassium (mEq/l)	5.6	3.3	4.8	4.9
Calcium (mg/dl)	11.6	–	–	–
Phosphorus (mg/dl)	4.7	–	–	–
C-reactive protein (mg/dl)	1	–	–	–

**Fig. 1:**
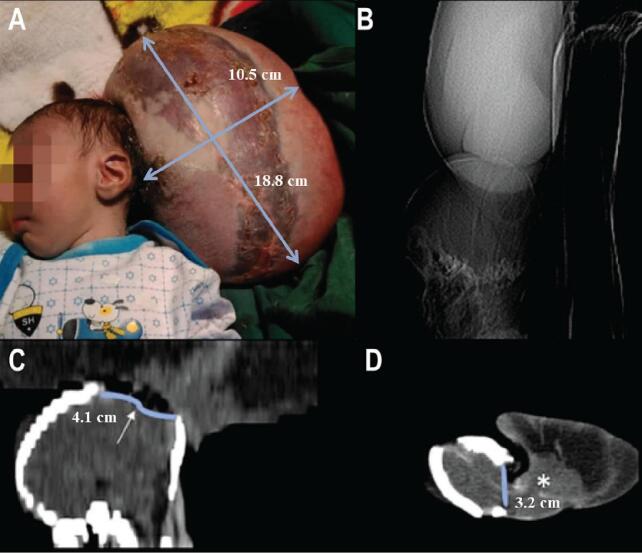
(A) Preoperative photograph showing a giant occipital encephalocele. (B) A preoperative plain radiograph shows a huge soft tissue mass. (C, D) Sagittal and coronal sections of the preoperative non-enhanced brain computed tomography scan. A bone defect is seen (blue line), with huge herniated meninges and brain tissues.

**Fig. 2:**
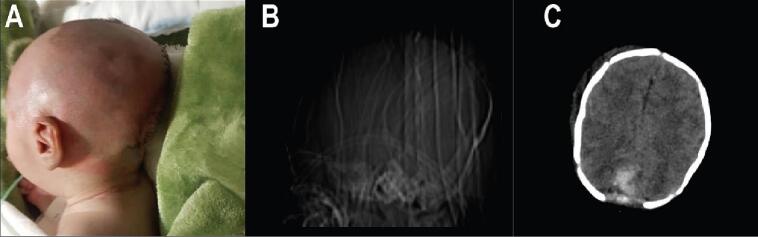
(A) Post-operative photo of the patients. (B) Post-operative plain radiograph showing removal of the lesion. (C) Axial section of the post-operative non-enhanced brain computed tomography scan. There is evidence of post-op change, including occipital bone defect, and also there is a hyperdense area at the site of surgery suggestive of hemorrhage.

After a brain computed tomography (CT) scan, the patient was scheduled for operation and resection of the giant occipital encephalocele (Fig. 1). General anesthesia was provided in the prone position. A circumferential incision was made on the mass, the margins of the skin were marked, and the encephalocele containing gliotic brain tissue and vessels was resected with the help of bipolar cautery. Dissection of the dural plane was performed around the bone defect of the occiput. Then, a primary dural repair was performed. Subcutaneous tissue and skin were sutured, and a dressing was applied. Extubation was done a few hours after surgery, and the patient was discharged with a satisfying condition. In 3-month, follow-up, the patient was well and had no complications (Fig. 2). And if the patient has acceptable developmental and cognitive milestones in the future, our team will consider cranioplasty surgery in the future.

## 3 Discussion

The most common type of encephalocele is occipital [3,10]. Its size can vary from small to large. In the giant form, the size of the encephalocele is larger than the head [3]. As our patient did, most encephaloceles have no known etiology [5]. Some teratogens may contribute to this condition, such as vitamin A, clofibrate, and sodium arsenate [11]. Hydrocephalus, Dandy-Walker syndrome, microcephaly, craniosynostosis, and Chiari malformation are anomalies linked to encephalocele. Therefore, it is essential to check for these conditions using appropriate imaging studies before starting any treatment or procedures [12,13]. Hydrocephalus and ventriculomegaly were absent in our case. Patients with encephaloceles and coexistent hydrocephalus have a far worse neurological outcome [13]; the absence of hydrocephalus in our patient may be a reason for the good neurological outcome.

Treating a giant encephalocele is challenging. We chose the prone position for the operation as it is the best for such operations, because it gave us full access to all the dimensions of the encephalocele, and its resection was not possible in a supine or lateral position. Also, in previous studies, the prone position was used [2,14]. Before incision, cerebrospinal fluid (CSF) aspiration can make sac dissection easier [3]. During the operation, the team must pay special attention to blood loss, hypothermia, hypoglycemia, precise protection of the endotracheal tube in the prone position, and complications associated with prolonged anesthesia [7,15]. Meningitis, CSF leakage, wound infection and dehiscence, and hydrocephalus are some examples of postoperative complications. [2]. Numerous elements could influence the prognosis, including the sac's size, the volume of neural tissue inside, any related abnormalities, and any complications following surgery. The amount of brain tissue in the sac is the most important prognostic factor [[16], [17], [18]]. In a study of 14 patients with giant encephalocele from 2002 to 2009 in India, 66% had good mental status after the operation [19]. In a more recent study by Velho et al., 54 patients were examined, and occipital encephalocele was observed in 14 cases and was rarer than the frontoethmoidal type. In addition, they found that CSF leakage is the most common complication seen in this surgery, but fortunately, our patient did not suffer from it. Finally, most of their patients did not need to close the bone defect, which was the case in our case [20].

## 4 Conclusion

Congenital giant occipital encephaloceles can be identified clinically shortly after birth. Because of their enormous size, they pose a tremendous surgical challenge. Surgical repair must be performed as early as possible.

## Ethics approval and consent to participate

Our study has been reviewed and approved by the Medical Ethics Committee xxx. The study is exempt from ethical approval in our institution.

## Funding

None.

## Author contribution

**Hamide Barzegar:** Conceptualization, Data Curation, Writing - Original Draft, Writing - Review & Editing. **Marzieh Davoodi:** Conceptualization, Investigation, Supervision, Writing - Original Draft, Writing - Review & Editing. **Shahnaz Pourarian:** Conceptualization, Data Curation, Writing - Original Draft, Writing - Review & Editing, Supervision. **Hamid Reihani:** Conceptualization, Data Curation, Writing - Original Draft, Writing - Review & Editing, Supervision, Project administration

## Guarantor

Corresponding author.

## Research registration number

None.

## Inform consent

Written informed consent was obtained from the patient's parents to publish this Case report and any accompanying images. A copy of the written consent is available for review and can be requested by the journal's editor at any time.

## Availability of data and materials

Patient data can be requested from the authors. Don't hesitate to contact the corresponding author if you are interested in such data.

## Conflict of interest statement

The authors declare that they have no competing interests.
